# An adjuvant formulation containing Toll-like Receptor 7 agonist stimulates protection against morbidity and mortality due to *Anaplasma marginale* in a highly endemic region of west Africa

**DOI:** 10.1371/journal.pone.0306092

**Published:** 2024-08-29

**Authors:** James E. Futse, Songliedong Zumor-Baligi, Charles N. K. Ashiagbor, Susan M. Noh, Christopher B. Fox, Guy H. Palmer

**Affiliations:** 1 Animal Disease Biotechnology Laboratory, Department of Animal Science, School of Agriculture, College of Basic and Applied Sciences, University of Ghana, Legon, Accra, Ghana; 2 Animal Diseases Research Unit, USDA-ARS, Pullman, Washington, United States of America; 3 Department of Veterinary Microbiology and Pathology, Washington State University, Pullman, Washington, United States of America; 4 Paul G. Allen School for Global Health, Washington State University, Pullman, Washington, United States of America; 5 Access to Advanced Health Institute, Seattle, Washington, United States of America; 6 Department of Global Health, University of Washington, Seattle, Washington, United States of America; 7 Institute for Tropical Infectious Diseases, University of Nairobi, Nairobi, Kenya; University of Missouri College of Veterinary Medicine, UNITED STATES

## Abstract

Efficient cattle production and provision of animal-sourced foods in much of Africa is constrained by vector-borne bacterial and protozoal diseases. Effective vaccines are not currently available for most of these infections resulting in a continuous disease burden that limits genetic improvement. We tested whether stimulation of innate immunity using the Toll-like Receptor (TLR) 7 agonist imiquimod, formulated with saponin and water-in-oil emulsion, would protect against morbidity and mortality due to *Anaplasma marginale*, a tick-borne pathogen of cattle highly endemic in west Africa. In Trial 1, haplotype matched Friesian x Sanga (F1) *A*. *marginale* negative calves were allocated to either the experimental group (n = 10) and injected with the synthetic TLR 7 agonist/saponin formulation or to an untreated control group (n = 10). TLR7 agonist/saponin injected calves responded with significantly elevated rectal temperature, enlarged regional lymph nodes, and elevated levels of IL-6 post-injection as compared to control group calves. All calves were then allowed to graze in pasture for natural exposure to tick transmission. All calves in both groups acquired *A*. *marginale*, consistent with the high transmission rate in the endemic region. The need for antibiotic treatment, using pre-existing criteria, was significantly lower in the experimental group (odds ratio for not requiring treatment was 9.3, p = 0.03) as compared to the control group. Despite treatment, 6/10 calves in the control group died, reflecting treatment failures that are typical of anaplasmosis in the acute phase, while mortality in the experimental group was 1/10 (odds ratio for survival was 13.5, p = 0.03). The trial was then repeated using 45 Friesian x Sanga calves per group. In Trial 2, the odds ratios for preventing the need for treatment and for mortality in the TLR7 agonist/saponin experimental group versus the control group were 5.6 (p = 0.0002) and 7.0 (p = 0.004), respectively, reproducing the findings of the initial trial. Together these findings demonstrate that innate immune stimulation using a TLR7 agonist formulated with saponin and water-in-oil emulsion provides significant protection against disease caused by tick borne *A*. *marginale* in highly susceptible cross-bred cattle, critically important for their potential to increase productivity for smallholder farmers in Africa.

## Introduction

Improved productivity of cattle in smallholder households in sub-Saharan Africa has been shown to increase milk available for consumption and sale and resulted in increased household expenditures on health and child education [1–3]. However, genetic improvement is limited by the enhanced susceptibility of higher producing cattle breeds to endemic vector-borne infectious disease as compared to lower producing indigenous breeds. As a result, smallholder farmers who lack the resources for disease prevention in improved breeds using acaricides/insecticides or treatment of sick animals are unable to improve milk production [4].

In west Africa, anaplasmosis, caused by the rickettsial pathogen *Anaplasma marginale*, is endemic with high prevalence and causes acute, usually mild disease in indigenous breeds but severe, often fatal, disease in improved cattle breeds [5–10]. Notably however, animals that recover from acute disease remain protected from subsequent disease despite repeated exposure [11, 12]. We hypothesized that stimulation of an innate immune response in higher productivity, genetically improved cross-breed cattle would provide protection against severe disease during the acute phase, thus allowing progression to long-term immunity. To test this hypothesis, we inoculated F1 Friesian-Sanga calves with a synthetic Toll-like Receptor (TLR) 7 agonist formulated with saponin and water-in-oil emulsion, assessed the innate immune responses, and then exposed the animals to natural tick-borne challenge within a highly endemic region of Ghana. Major Histocompatibility Locus (MHC) haplotype matched control calves were exposed together with the experimental group. Following confirmation of naturally transmitted *A*. *marginale* infection in all calves, protection from severe disease that required antibiotic treatment and mortality was assessed and compared between the two groups. The trial was then repeated for reproducibility. Here we report the outcome of these experimental trials and discuss the results in the context of stimulating innate immunity to allow genetic improvement of cattle in sub-Saharan Africa.

## Materials and methods

### TLR agonist formulation

Synthetic TLR agonist imiquimod stimulates TLR7 [13–16] and has been shown to bind and stimulate the cognate receptor on bovine cells. For our set of experiments, 1 mg of TLR7 agonist (imiquimod, Chemagis) and 1 mg of semi-purified saponins from *Quillaja saponaria* (VetSap, Desert King) were delivered in 1 ml of a water-in-oil emulsion containing 53.5% v/v mineral oil (USP grade, Spectrum Chemical), 6.5% v/v isostearic acid (TCI), 0.97% w/v mannide monooleate (Sigma Aldrich), 0.2% w/v cholesterol (J.T. Baker), and phosphate buffered saline (Gibco) prepared by high shear mixing followed by gamma irradiation at the Access to Advanced Health Institute (formerly known as the Infectious Diseases Research Institute, Seattle, WA USA). Immediately before use, the formulation was resuspended by vigorous shaking.

### Source of crossbred calves and MHC DRB3 haplotypes

Three-month old Friesian x Sanga (F1) calves, seronegative for *A*. *marginale* by Msp5 CI-ELISA [17], were obtained from Amrahia Dairy Farm and Animal Research Institute of the Council for Scientific and Industrial Research of Ghana. To control for potential confounding effects of MHC haplotype on the immune responses [18–20], calves were MHC haplotyped and all were confirmed to express DRB3*2404, DRB3*2711, DRB3*2405, DRB3*2406, and DRB3*2407, alleles common within the Sanga breed from Ghana. For trial 1, three-month old Friesian x Sanga calves were allocated randomly to two equal groups. The experimental group (n = 10) was injected with the TLR agonist/saponin emulsion and the control group (n = 10) was left untreated. For trial 2, the experiment was replicated using 45 Friesian x Sanga calves per group.

### Animal care and use

Calves in the experimental group were inoculated subcutaneously with 1 ml of TLR7 agonist formulated with saponin and water-in-oil emulsion. Blood collection by jugular venipuncture from calves in both groups was <10 ml (approximately 0.2% blood volume) per day for eight days pre- and post-injection and then weekly. Neither inoculation or venipuncture requires analgesia or anesthesia. Calves were allowed to graze on pasture by day, during which time there was natural exposure to ticks (i.e. there was no deliberate feeding of ticks on animals nor deliberate infection with *A*. *marginale*). All calves were examined daily and oxytetracycline treatment was instituted immediately following the onset of *a priori* defined clinical parameter of anemia in order to relieve suffering. Treated calves were separated from the group and monitored twice daily. No animals were intentionally sacrificed during the study. The cattle used in this study were treated in strict accordance with guidelines set by University of Ghana Institutional Animal Care and Use Committee and the protocol for blood sampling was approved by the Noguchi Memorial Institute for Medical Research (NIACUC protocol number: 2015-01-5X).

### Measurement of induced innate response

The rectal temperature and the sizes of pre-scapular lymph node of each calf were examined daily for four days prior to injection with the TLR agonist/saponin emulsion and for the control group calves to record pre-treatment parameters. Subsequently, 1ml of the agonist preparation was injected subcutaneously into calves (n = 10) in the experimental group. Calves (n = 10) in the control group were not injected. The rectal temperature and sizes of the lymph nodes from individual calves were measured, at same time (08:00 hours), daily for additional four days post-inoculation. Blood was collected daily and serum levels of the proinflammatory cytokine IL-6 were measured by capture cytokine-specific antibody ELISA (NEO Scientific Biolab, Cambridge, Massachusetts) and a standard curve constructed using the SoftMax^®^ Pro 6 Software. The data were shown as the mean (+/- standard deviation) for all pre- and post-inoculation timepoints for individual calves in each group and reported at the group level as mean (+/- standard error). Significance between the TLR agonist/saponin emulsion injected and control groups was determined by the Student’s *t*-test with Holm-Sidak correction for multiple comparisons.

### Detection of infection

Screening for *A*. *marginale* infection was done using *msp5* PCR of peripheral blood and by Msp5 CI-ELISA to detect seroconversion [17, 21–23].

### Antibiotic treatment

All calves were observed clinically twice daily and packed cell volume (PCV) determined. If PCV decreased to ≤ 28, treatment with 20mg/kg long-acting tetracycline was initiated and the animal moved to a separate pen. Treatment was repeated twice at 48-hour intervals.

### Weight gain

Calves in Trial 1 were weighed at day 0, day 28 and 48 to measure effects of stimulation of innate immunity and acute *A*. *marginale* infection. A subset of calves from Trials 1 and 2 (n = 10 per group) were maintained on pasture for 13 months to determine if there were any long-term adverse effects on weight gain. Calves were weighed at 28-day intervals in the morning before they were taken out for grazing and the mean monthly gain determined.

## Results

### Synthetic TLR7 agonist/saponin emulsion injection stimulates innate immunity of crossbred calves

In Trial 1, the rectal temperature from each calf was recorded each day for four days post-inoculation and compared to the same period prior to TLR7 agonist/saponin injection. This controls for individual variation among the calves. Similarly, the temperatures were collected on the same days and same time for the non-inoculated control calves. Calves in the TLR7 agonist/saponin emulsion experimental group developed significantly increased temperatures following injection (increase of 0.98°C ± 0.08°C; p = 0.00003) as compared to pre-inoculation temperatures (Table 1 and S1 Table), while there was no significant change for the control calves (0.19°C ± 0.10°C; p = 0.49). The mean rectal temperature for the experimental group during the four days following TLR7 agonist/saponin injection was significantly higher than that of the control group over the same period (p = 0.001).

**Table 1:** Mean [±SD] rectal temperature of calves 4-days prior to and 4-days after inoculation with the TLR7 agonist/saponin emulsion.

	Pre-inoculation		Post-inoculation		Difference
**Crossbred calf #**	**Control Group** ^a^				
5NS24	38.6	[0.05]	38.8	[0.01]	0.2
M9	38.6	[0.07]	38.3	[0.13]	-0.3
3NS51	38.7	[0.05]	38.4	[0.04]	-0.3
3NS2	38.6	[0.05]	38.7	[0.25]	0.1
3NS53	38.6	[0.10]	38.8	[0.05]	0.2
M3768	38.5	[0.05]	38.8	[0.10]	0.3
M96	38.2	[0.21]	38.5	[0.25]	0.3
N1299	38.1	[0.10]	38.9	[0.15]	0.8
R270	38.2	[0.05]	38.3	[0.03]	0.1
R286	38.4	[0.13]	38.7	[0.02]	0.3
			**Mean ±SE**		**0.19 ±0.10**
	**Experimental Group**				
O554	38.7	[0.21]	39.5	[0.05]	0.8
4NS26	38.3	[0.05]	39.7	[0.13]	1.4
4NS22	38.4	[0.1]	39.6	[0.07]	1.2
3777	38.7	[0.04]	39.3	[0.23]	0.6
4NS21	38.6	[0.32]	39.3	[0.2]	0.7
5NS6	38.4	[0.07]	39.4	[0.11	1.0
4533	38.2	[0.05]	39.4	[0.31]	1.2
R287	38.7	[0.08]	39.5	[0.17]	0.8
N39	38.6	[0.2]	39.4	[0.22]	0.8
R288	38.6	[0.05]	39.9	[0.13]	1.3
			**Mean ±SE**		**0.98 ±0.08**

^**a**^ Control group calves that did not receive the TLR7 agonist/saponin emulsion were monitored over the same time period. The data shown are the means (+/- SD) of all daily timepoints for individual calves in each group. Calves in the TLR7 agonist/saponin emulsion experimental group developed significantly increased temperatures following injection (p = 0.00003) as compared to pre-inoculation temperatures. There was no significant change in the control group calves over the same period (p = 0.49), The mean rectal temperature for the experimental group during the four days following TLR7 agonist/saponin injection was significantly higher than that of the control group over the same period (p = 0.001).

Similarly, there were significant increases in the diameter of the pre-scapular lymph node in all experimental calves (Table 2 and S2 Table). The increase was significantly higher over the four days following TLR7 agonist/saponin emulsion injection as compared to the four days prior (p = 0.00001) and significantly greater than the diameter of the lymph nodes of the control calves (p = 0.0001), which did not increase in size (p = 0.28).

**Table 2:** Mean [±SD] diameter (mm) of the pre-scapular lymph nodes of calves 4-days prior to and 4-days after inoculation with the TLR7 agonist/saponin emulsion.

	Pre-inoculation		Post-inoculation		Difference
**Crossbred calf #**	**Control Group** ^a^				
5NS24	93.1	[1.0]	92.5	[0.2]	-0.6
M9	80.5	[0.8]	83.3	[0.8]	2.8
3NS51	93	[0.9]	92.6	[0.9]	-0.4
3NS2	79.2	[1.7]	80.1	[1.4]	0.9
3NS53	79.3	[1.1]	81.0	[1.3]	1.7
M3768	92.7	[1.3]	96.6	[0.7]	3.9
M96	92.2	[1.2]	92.6	[0.9]	0.4
N1299	78.3	[0.5]	81.5	[1.2]	3.2
R270	79.3	[1.6]	81.2	[0.8]	1.9
R286	79.7	[1.4]	83.3	[0.6]	3.6
			**Mean ±SE**		**1.72 ±0.48**
	**Experimental Group**				
O554	89.4	[0.7]	98.7	[2.5]	9.3
4NS26	78.8	[0.4]	99.4	[2.4]	20.6
4NS22	86.3	[1.5]	98.3	[1.1]	12.0
3777	76.6	[0.8]	90.1	[2.3]	13.5
4NS21	85.6	[0.9]	96.7	[2.2]	11.1
5NS6	84.4	[1.1]	96.6	[0.3]	12.2
4533	72.2	[0.8]	85.4	[1.3]	13.2
R287	75.2	[1.3]	98.1	[2.2]	22.9
N39	72.7	[0.5]	97.1	[2.7]	24.4
R288	71.8	[0.9]	89.0	[3.1]	17.2
			**Mean ±SE**		**15.64±1.7**

^a^ Control group calves that did not receive the TLR7 agonist/saponin emulsion were monitored over the same time period. The data shown are the means (+/- SD) of all daily timepoints for individual calves in each group. Calves in the TLR7 agonist/saponin emulsion experimental group developed significantly larger lymph nodes following injection (p = 0.00001) as compared to pre-inoculation lymph node diameter. There was no significant change in the control group calves over the same period (p = 0.28), The mean lymph node diameter for the experimental group during the four days following TLR7 agonist/saponin injection was significantly greater than that of the control group over the same period (p = 0.0001).

Consistent with the innate inflammatory response indicated by the increases in rectal temperature and lymph node size, IL-6 levels significantly increased (p = 0.00001) over the four-day period in the TLR7 agonist/saponin emulsion group as compared to the control group (Table 3 and S3 Table).

**Table 3:** Mean [±SD] IL-6 levels (log_10_) in serum of calves of calves 4-days prior to and 4-days after inoculation with the TLR7 agonist/saponin emulsion.

	Pre-inoculation		Post-inoculation		Difference
**Crossbred calf #**	**Control Group** ^a^				
5NS24	2.9	[0.2]	3.0	[0.7]	0.1
M9	1.8	[0.5]	2.1	[0.4]	0.3
3NS51	1.9	[0.7]	2.1	[0.7]	0.2
3NS2	2.3	[0.3]	2.5	[0.6]	0.2
3NS53	2.0	[06]	2.2	[0.2]	0.2
M3768	2.5	[0.2]	2.6	[0.8]	0.1
M96	2.1	[0.5]	2.3	[0.3]	0.2
N1299	2.1	[0.1]	2.4	[0.9]	0.3
R270	2.1	[0.8]	2.3	[0.9]	0.2
R286	1.9	[0.4]	2.4	[0.2]	0.5
			**Mean ±SE**		**0.23 ±0.04**
	**Experimental Group**				
O554	3.9	[1.2]	9.5	[1.9]	5.6
4NS26	1.6	[0.9]	5.5	[1.7]	3.9
4NS22	1.3	[2.6]	3.6	[0.3]	2.3
3777	3.4	[1.9]	4.1	[1.2]	0.7
4NS21	1.7	[1.9]	4.2	[1.6]	2.5
5NS6	1.1	[2.3]	7.4	[0.7]	6.3
4533	2.9	[1.3]	6.3	[0.7]	3.4
R287	2.8	[2.4]	3.5	[1.1]	0.7
N39	2.3	[0.8]	5.5	[0.8]	3.2
R288	2.5	[2.0]	4.6	[1.5]	2.1
			**Mean ±SE**		**3.07±0.58**

^a^ Control group calves that did not receive the TLR7 agonist/saponin emulsion were monitored over the same time period. The data shown are the means (+/- SD) of all daily timepoints for individual calves in each group. Calves in the TLR7 agonist/saponin emulsion experimental group had significantly higher IL-6 levels (p = 0.00001) over the four-day post-inoculation period as compared to the control group.

### Synthetic TLR7 agonist/saponin injection reduces subsequent need for antibiotic treatment and mortality of crossbred calves following *A*. *marginale* infection

In Trial 1, all calves tested positive by PCR and Msp5 CI-ELISA for *A*. *marginale* within 22 days of exposure to field challenge. This was expected given the high levels of transmission in Ghana [7]. Calves were monitored daily and antibiotic treatment was initiated if PCV decreased to 28% or below. Of the control group (n = 10) that did not receive the TLR7 agonist/saponin emulsion, 8 calves met the stated criteria for antibiotic treatment while 3 calves in the TLR7 agonist/saponin emulsion injected group required antibiotics (Table 4). The mean low PCV was significantly greater in the experimental group as compared to the control group (Table 4). Despite antibiotic treatment, 6 calves in the control group died as compared to 1 in the experimental group (Table 4). Based on these results, the trial was then independently repeated using larger group sizes, n = 45 for each group.

**Table 4:** Trial 1: Mean PCV, antibiotic treatment, and mortality for stimulated and control calves.

Group	Mean PCV± SE (95% CI)	Antibiotic Treatment	Mortality
Stimulated (n = 10)	28.9 ± 3.3 (26.5–31.3)	3/10	1/10
Control (n = 10)	24.78 ± 3.4 (22.3–27.2)	8/10	6/10

PCV, packed cell volume; SE, standard error; CI, confidence intervals.

The PCV data shown are the means (+/- SE) for all calves in each group.

Difference in PCV between stimulated and control group, p<0.0001. Odds ratio for not requiring antibiotic treatment for the stimulated group versus control group was 9.3 (95% confidence interval 1.2–72.9; p = 0.03). Odds ratio for survival was 13.5 (95% confidence interval 1.9–152.2; p = 0.03) for the stimulated group as compared to the control group.

In trial 2, *A*. *marginale* infection was again confirmed in all calves within three weeks of natural exposure. Of the 45 calves in the control group, 34 calves (76%) met antibiotic treatment criteria while 16 of 45 (36%) in the TLR7 agonist/saponin injected group required treatment (Table 5). In the control group, 15 calves died, despite treatment, while in the experimental group 3 died (Table 5).

**Table 5:** Trial 2: Mean PCV, antibiotic treatment, and mortality for stimulated and control calves.

Group	Mean PCV± SE (95% CI)	Antibiotic Treatment	Mortality
Stimulated (n = 45)	27.1 ± 4.7 (23.7–30.5)	16/45	3/45
Control (n = 45)	23.7 ± 3.7 (21.1–26.3)	34/45	15/45

PCV, packed cell volume; SE, standard error; CI, confidence intervals.

The PCV data shown are the means (+/- SE) for all calves in each group.

Difference in PCV between stimulated and control group, p<0.0001. Odds ratio for not requiring antibiotic treatment for the stimulated group versus control group was 5.6 (95% confidence interval 2.2–14; p = 0.0002). Odds ratio for survival was 7.0 (95% confidence interval 1.9–26.3; p = 0.004) for the stimulated group as compared to the control group.

### IL-6 levels correlate with protection against anemia due to *A*. *marginale* infection

**Fig 1:**
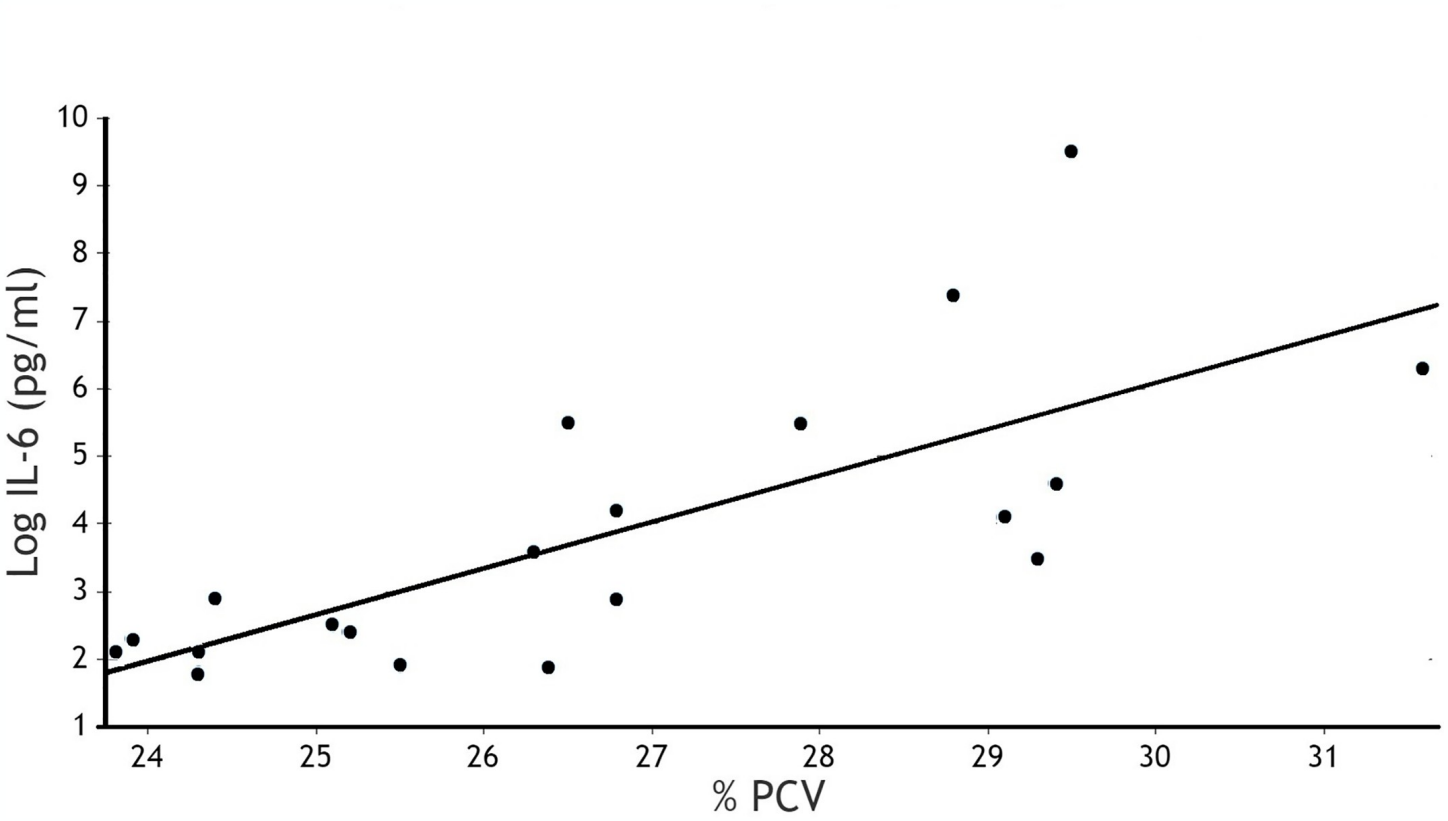
Correlation between IL-6 levels and PCV of calves. The X values represent the mean percent packed cell volume (% PCV) of all 20 calves (n = 10 for control and 10 for calves injected with the TLR7 agonist/saponin emulsion. The Y values represent the corresponding mean log IL-6 (pg/ml) of individual calves. R is: 0.742; P = 0.0001; R^2^ = 0.55.

The mean IL-6 levels were plotted against the mean PCV level of each calf in both groups from Trial 1 (Fig 1 and S4 Table). There was a statistically significant correlation (r = 0.74; p<0.0001) between IL-6 levels and anemia, at the individual calf level, regardless of the group.

### Synthetic TLR agonist/saponin emulsion injection does not interfere with the long-term weight gain of calves

We also examined whether TLR7 agonist/saponin emulsion injection and short-term stimulation of the innate immune response had a long-term impact on weight gain. The average weight gain of calves injected with the TLR7 agonist/saponin in Trials 1 and 2 was 1.72kg ± /month compared with 1.67kg ± /month for the control group. The overall body weight at the end of the trial was not significantly different (p = 0.38) between the two groups.

## Discussion

*Anaplasma marginale* is highly endemic in Ghana, throughout sub-Saharan Africa, and in most tropical and subtropical countries [7, 21, 22]. Severe morbidity and mortality occur during the acute phase of infection, when bacteremia levels exceed 10^9^ per ml. Notably, animals that survive acute infection develop life-long immunity [11, 12]. As a result, two strategies have been used to manage disease impact. The first is the use of a live vaccines, which involves deliberate infection of cattle with a less virulent subspecies *A*. *marginale* ss. *centrale* [23–26]. Although generally effective, the widespread use of the live vaccine has been constrained by the requirement for a cold chain based on preservation in liquid nitrogen, which is difficult and expensive to ensure in low-income tropical countries. The second approach has been to rely on indigenous breeds of cattle which, although equally susceptible to infection, are significantly more resistant to developing severe morbidity and mortality [27–33]. Although effective, the inability to introduce higher productivity traits into indigenous breeds by crossbreeding without increased susceptibility to severe morbidity and mortality has resulted in lower productivity and decreased economic gains for smallholder farmers. Relevant to the cross-bred cattle used in this current study, F1 Sanga x Friesian cows provide approximately twice the daily milk yield as compared to Sanga cows raised under the same pasture conditions [34–36]. Even minor increases in milk production have been shown to translate into increased household income and expenditures on additional food, health care, and education [2, 37]. Based on the concept that there is an energetic trade-off between resources dedicated to innate immunity versus productivity, we tested the hypothesis that direct stimulation of the innate immune system would provide sufficient short-term protection in crossbred cattle to avoid severe morbidity and mortality. The selection of the synthetic TLR7 agonist imiquimod and its incorporation into a slow-release water-in-oil emulsion was based on the evidence that bovine TLRs 7 and 8 responded to stimulation with cytokine release [13, 14, 16, 38, 39]. The confirmation that F1 Sanga x Friesian crossbred cattle responded with significantly increased temperatures, enlarged regional lymph nodes, and IL-6 levels over the first four days post-injection indicated innate immune stimulation. The subsequent significant reduction in need for antibiotic treatment and reduced mortality, confirmed in two independent trials with a total of 55 animals per group, support the efficacy of this approach. Notably, tracking subsequent weight gain of animals in the second trial group revealed no significant difference between the TLR7 agonist/saponin emulsion injected and control cattle, indicating that any energetic trade-off between innate immunity and productivity was transient. This latter finding is important as the overall goal is to increase productivity for the smallholder farmer.

This study is a proof of concept that supports an overall approach to managing severe disease due to *A*. *marginale* infection. There are several important caveats. The first is that although there was a statistically significant reduction in the need for antibiotic treatment and in mortality, multiple animals in the TLR7 agonist/saponin injected groups required treatment and 7% died (versus 38% in the controls). We did not include a control group of age-matched Sanga cattle that would have allowed us to determine if the TLR agonist/saponin induced a similar level of protection as fully indigenous cattle, which would be a relevant benchmark. Secondly, the selection of agonist was largely empirical based on the ability of TLR 7 agonist/saponin stimulation to increase CD4+ T cell secretion of IFN-γ [40], previously show to associate with immune protection against *A*. *marginale* challenge [41]. Similarly, the dose and formulation were also empirical and unlikely to be optimal. However, the correlation between protection from anemia as a measure of disease severity and IL-6 levels suggests that IL-6 is a measurable parameter to optimize agonist, dose and formulation. It is notable that the components employed in the formulation are low cost and commercially available, and the processing requires minimal equipment. Additional or different agonists, doses, or slow-release formulation should be tested with the goal of reducing antibiotic treatment and mortality to less than or equal to that of the fully indigenous Sanga. In addition, the duration of innate immunity and protection induced by the TLR7 agonist/saponin is unknown. In the current study conducted in an endemic region with a high level of transmission, challenge occurred within three weeks following natural exposure for which short-term protection may be sufficient. In regions with lower transmission or where transmission is seasonal, the duration of protection becomes a key question and requires additional experimentation. Finally, efficacy was only tested against acute *A*. *marginale* infection. However, there are multiple infectious diseases of tropical and subtropical livestock, including babesiosis and East Coast Fever, that have a similar course of acute disease followed by life-long antigen specific immunity; the approach of short-term stimulation of innate immunity may be an effective strategy for protection against multiple pathogens.

## Supporting information

S1 Table Rectal temperature of calves. The rectal temperatures of individual calves in the control and experimental (TLR agonist) groups four days prior to and four days following injection.
(DOCX)

S2 Table Lymph node sizes. The diameter of prescapular lymph nodes of individual calves in the control and experimental (TLR agonist) groups four days prior to and four days following injection.
(DOCX)

S3 Table IL-6 levels in individual calves. The IL6 levels (pg/ml) of individual calves in the control and experimental (TLR agonist) groups 24, 48, and 72 hours following injection.
(DOCX)

S4 Table Mean IL-6 levels and mean PCV in individual calves. The mean IL6 levels (pg/ml) and the mean PCV (%) of individual calves in the control and experimental (TLR agonist) groups.
(DOCX)
