# Supplementary material for: An adjuvant formulation containing Toll-like Receptor 7 agonist stimulates protection against morbidity and mortality due to Anaplasma marginale in a highly endemic region of west Africa
Source: PLoS One. 2024 Aug 29;19(8):e0306092. doi: 10.1371/journal.pone.0306092 (PMC11361566; doi:10.1371/journal.pone.0306092)
Supplement: S1 Table — The rectal temperatures of individual calves in the control and experimental (TLR agonist) groups four days prior to and four days following injection. (DOCX) [file pone.0306092.s001.docx]

| **S1 Table.** | |  | **RECTAL TEMPERATURE OF CALVES/^O^C** | | | | |  |  |  |
| --- | --- | --- | --- | --- | --- | --- | --- | --- | --- | --- |
|  | Calf ID | **4 Days prior to stimulation** | | | |  | **4 Days after stimulation** | | |  |
| Control group | | **Day1** | **Day 2** | **Day 3** | **Day 4** | FFFF | **Day1** | **Day 2** | **Day 3** | **Day 4** |
|  | **5NS24** | 38.7 | 38.6 | 38.7 | 38.6 |  | 38.7 | 38.9 | 38.9 | 38.6 |
|  | **M9** | 38.6 | 38.6 | 38.6 | 38.6 |  | 38.7 | 38.6 | 38.7 | 38.6 |
|  | **3NS51** | 38.6 | 38.7 | 38.7 | 38.7 |  | 38.3 | 38.6 | 38.4 | 38.4 |
|  | **3NS2** | 38.7 | 38.6 | 38.6 | 38.4 |  | 38.6 | 38.8 | 38.9 | 38.5 |
|  | **3NS53** | 38.6 | 38.8 | 38.5 | 38.6 |  | 38.7 | 38.8 | 38.6 | 38.7 |
|  | **M3768** | 38.5 | 38.6 | 38.6 | 38.6 |  | 38.9 | 38.8 | 38.6 | 38.7 |
|  | **M96** | 38.7 | 38 | 38 | 38 |  | 38.6 | 38.3 | 38.6 | 38.6 |
|  | **N1299** | 38 | 38 | 38.2 | 38.3 |  | 37.9 | 37.9 | 38.4 | 38.8 |
|  | **R270** | 38.7 | 38 | 38.2 | 38 |  | 38.5 | 38.7 | 38.6 | 38.6 |
|  | **R286** | 37.9 | 38.6 | 38.6 | 38.6 |  | 38.6 | 38.6 | 38.6 | 38.7 |
|  |  |  |  |  |  |  |  |  |  |  |
| Experimental | |  |  |  |  |  |  |  |  |  |
|  | **O554** | 38.7 | 38.6 | 38.8 | 38.6 |  | 39.2 | 39.5 | 39.9 | 39.3 |
|  | **4NS26** | 38.2 | 38.2 | 38.5 | 38.4 |  | 39.7 | 39.3 | 39.8 | 39.9 |
|  | **4NS22** | 38.2 | 38.6 | 38.5 | 38.4 |  | 39.5 | 39.6 | 39.5 | 39.7 |
|  | **3777** | 38.7 | 38.8 | 38.9 | 38.6 |  | 39.2 | 39.8 | 39.6 | 38.7 |
|  | **4NS21** | 38.4 | 38.9 | 38.4 | 38.7 |  | 39.6 | 39.6 | 39.2 | 38.9 |
|  | **5NS6** | 39.1 | 38.7 | 38.6 | 38.6 |  | 39.7 | 39.6 | 39.2 | 39 |
|  | **4533** | 38.8 | 38.7 | 38.7 | 38.6 |  | 39.6 | 39.4 | 39.7 | 38.7 |
|  | **R287** | 39 | 38.8 | 38.6 | 38.6 |  | 39.3 | 39.9 | 39.5 | 38.8 |
|  | **N39** | 38.7 | 38.5 | 38.6 | 38.6 |  | 39.2 | 39.8 | 39.7 | 39 |
|  | **R288** | 38.6 | 38.5 | 38.7 | 38.5 |  | 39.8 | 40.2 | 39.9 | 39.7 |
