# Supplementary material for: An adjuvant formulation containing Toll-like Receptor 7 agonist stimulates protection against morbidity and mortality due to Anaplasma marginale in a highly endemic region of west Africa
Source: PLoS One. 2024 Aug 29;19(8):e0306092. doi: 10.1371/journal.pone.0306092 (PMC11361566; doi:10.1371/journal.pone.0306092)
Supplement: S2 Table — The diameter of prescapular lymph nodes of individual calves in the control and experimental (TLR agonist) groups four days prior to and four days following injection. (DOCX) [file pone.0306092.s002.docx]

|  | **S2 Table.** | |  | **SIZES OF PRESCAPULAR LYNODES[mm] OF CALVES** | | | | | | | |  |
| --- | --- | --- | --- | --- | --- | --- | --- | --- | --- | --- | --- | --- |
|  | Calf ID | **Before stimulation with TLR7** | | | | | |  | **After stimulation with TL7** | | | |
| Control group | | **Day1** | **Day 2** | | **Day 3** | **Day 4** | |  | **Day1** | **Day 2** | **Day 3** | **Day 4** |
|  | **5NS24** | 92.9 | 92.6 | | 93.2 | 93.6 | |  | 92.8 | 92.3 | 92.2 | 92.6 |
|  | **M9** | 80.1 | 81.5 | | 79.2 | 80.3 | |  | 81.3 | 83.7 | 83.9 | 84.4 |
|  | **3NS51** | 93.1 | 93.2 | | 92.9 | 92.8 | |  | 93 | 92.8 | 92.1 | 92.4 |
|  | **3NS2** | 79.1 | 79.5 | | 79.3 | 79 | |  | 79.8 | 80.1 | 80.4 | 80.2 |
|  | **3NS53** | 79.5 | 79.2 | | 79.6 | 79.1 | |  | 79.7 | 80.4 | 81 | 82.8 |
|  | **M3768** | 92.3 | 92.6 | | 92.8 | 93 | |  | 94 | 96.9 | 97.7 | 97.9 |
|  | **M96** | 92.1 | 91.9 | | 92.3 | 92.4 | |  | 92.7 | 93 | 91.8 | 91.2 |
|  | **N1299** | 78.4 | 78.7 | | 77.9 | 78.1 | |  | 79.9 | 82 | 82.3 | 81.9 |
|  | **R270** | 79.2 | 79.2 | | 79.7 | 79.2 | |  | 80.8 | 81.5 | 81.6 | 81 |
|  | **R286** | 79.5 | 78.9 | | 80.1 | 80.2 | |  | 82.3 | 83.6 | 83.4 | 83.8 |
|  |  |  |  | |  |  | |  |  |  |  |  |
| Experimental | |  |  | |  |  | |  |  |  |  |  |
|  | **O554** | 89.1 | 89.6 | | 89.7 | 89.3 | |  | 98.1 | 99.3 | 99 | 98.5 |
|  | **4NS26** | 78.9 | 78.4 | | 78.7 | 79.1 | |  | 93.7 | 102.1 | 99.4 | 99.8 |
|  | **4NS22** | 86.1 | 86.6 | | 86.2 | 86.4 | |  | 97 | 99 | 99.1 | 98.2 |
|  | **3777** | 77.1 | 76.2 | | 76.5 | 76.7 | |  | 89 | 92.3 | 90 | 89 |
|  | **4NS21** | 85.5 | 85.3 | | 85.7 | 86 | |  | 94.8 | 99 | 97.1 | 95.9 |
|  | **5NS6** | 84.2 | 84.5 | | 84.7 | 84.3 | |  | 90.1 | 104.1 | 97.6 | 94.7 |
|  | **4533** | 72.1 | 71.8 | | 72 .4 | | 72.6 |  | 78.3 | 98.2 | 84 | 81.2 |
|  | **R287** | 75.1 | 75.3 | | 75.0 | 75.3 | |  | 90.6 | 114.3 | 94.4 | 93.2 |
|  | **N39** | 72.7 | 72.6 | | 72.4 | 73 | |  | 93.6 | 102.3 | 97.4 | 95.2 |
|  | **R288** | 71.9 | 71.6 | | 72.1 | 71.7 | |  | 95.3 | 105.2 | 99.4 | 96.2 |
