# Supplementary material for: An adjuvant formulation containing Toll-like Receptor 7 agonist stimulates protection against morbidity and mortality due to Anaplasma marginale in a highly endemic region of west Africa
Source: PLoS One. 2024 Aug 29;19(8):e0306092. doi: 10.1371/journal.pone.0306092 (PMC11361566; doi:10.1371/journal.pone.0306092)
Supplement: S3 Table — The IL6 levels (pg/ml) of individual calves in the control and experimental (TLR agonist) groups 24, 48, and 72 hours following injection. (DOCX) [file pone.0306092.s003.docx]

| **S3 Table.** | |  |  |  |  |  |
| --- | --- | --- | --- | --- | --- | --- |
| **IL-6 levels in individual cattle** | | | |  |  |  |
| CALF ID | TIME/HR | **INJECTED** | | CALF ID | **CONTROL** | |
| O554 | **24** | 4.173 |  | 5NS24 | 1.789 |  |
|  | **48** | **9.524** |  |  | 1.895 |  |
|  | **72** | 4.19 |  |  | 2.316 |  |
| 4NS26 | **24** | 2.769 |  | M9 | 2.421 |  |
|  | **48** | **5.504** |  |  | 2.526 |  |
|  | **72** | 3.744 |  |  | 2.927 |  |
| 4NS22 | **24** | 2.79 |  | 3NS51 | 1.789 |  |
|  | **48** | **3.501** |  |  | 1.895 |  |
|  | **72** | 2.632 |  |  | 2.316 |  |
| 3777 | **24** | 2.541 |  | 3NS2 | 2.421 |  |
|  | **48** | **4.007** |  |  | 2.526 |  |
|  | **72** | 2.679 |  |  | 2.927 |  |
| 4NS21 | **24** | 3.823 |  | 3NS53 | 1.789 |  |
|  | **48** | 4.173 |  |  | 1.895 |  |
|  | **72** | 2.914 |  |  | 2.316 |  |
| 5NS6 | **24** | 4.094 |  | 3768 | 2.421 |  |
|  | **48** | 7.362 |  |  | 2.526 |  |
|  | **72** | 4.349 |  |  | 2.927 |  |
| 4533 | **24** | 4.195 |  | 96 | 2.127 |  |
|  | **48** | 6.294 |  |  | 2.083 |  |
|  | **72** | 3.744 |  |  | 1.907 |  |
| R287 | **24** | 2.795 |  | N1299 | 1.015 |  |
|  | **48** | 5.204 |  |  | 1.625 |  |
|  | **72** | 2.978 |  |  | 1.249 |  |
| 39 | **24** | 3.403 |  | R270 | 2.378 |  |
|  | **48** | 5.497 |  |  | 1.699 |  |
|  | **72** | 2.679 |  |  | 1.099 |  |
| R288 | **24** | 2.982 |  | R286 | 2.859 |  |
|  | **48** | 4.632 |  |  | 2.751 |  |
|  | **72** | 2.723 |  |  | 2.312 |  |
