# Supplementary material for: An adjuvant formulation containing Toll-like Receptor 7 agonist stimulates protection against morbidity and mortality due to Anaplasma marginale in a highly endemic region of west Africa
Source: PLoS One. 2024 Aug 29;19(8):e0306092. doi: 10.1371/journal.pone.0306092 (PMC11361566; doi:10.1371/journal.pone.0306092)
Supplement: S4 Table — The mean IL6 levels (pg/ml) and the mean PCV (%) of individual calves in the control and experimental (TLR agonist) groups. (DOCX) [file pone.0306092.s004.docx]

S4 Table : Levels of IL-6 readouts in calves and the corresponding percent Packed Cell Volume [PCV].
